# Hypertensive Disorders: Prevalence, Perinatal Outcomes and Cesarean Section Rates in Pregnant Women Hospitalized for Delivery

**DOI:** 10.1055/s-0040-1714134

**Published:** 2020-11-30

**Authors:** Francisco Lírio Ramos Filho, Carlos Maurício de Figueiredo Antunes

**Affiliations:** 1 Santa Casa de Belo Horizonte, Belo Horizonte, MG, Brazil

**Keywords:** pre-eclampsia, prevalence, cesarean section, preterm infant, low birthweight infant, pré-eclâmpsia, prevalência, cesárea, recém-nascido pré-termo, recém-nascido de baixo peso

## Abstract

**Objective** To evaluate the prevalence of hypertensive disorders, perinatal outcomes (preterm infants, low birthweight infants and Apgar score < 7 at the 5th minute and fetal deaths) and the cesarean rates in pregnant women hospitalized for delivery at the Maternidade Hilda Brandão da Santa Casa de Belo Horizonte, Belo Horizonte, state of Minas Gerais, Brazil, from March 1, 2008 to February 28, 2018.

**Methods** A case-control study was performed, and the groups selected for comparison were those of pregnant women with and without hypertensive disorders. Out of the 36,724 women, 4,464 were diagnosed with hypertensive disorders and 32,260 did not present hypertensive disorders

**Results** The prevalence of hypertensive disorders was 12.16%; the perinatal outcomes and cesarean rates between the 2 groups with and without hypertensive disorders were: preterm infants (21.70% versus 9.66%, odds ratio [OR] 2.59, 95% confidence interval [CI], 2.40–2.80, *p* < 0.001); low birthweight infants (24.48% versus 10.56%; OR 2.75; 95% CI, 2.55–2.96; *p* < 0.001); Apgar score < 7 at the 5 ^th^ minute (1.40% versus 1.10%; OR 1.27; 95% CI, 0.97–1.67; *p* = 0.84); dead fetuses diagnosed prior to delivery (1.90% versus 0.91%; OR 2.12; 95% CI, 1.67–2.70; *p* < 0.001); cesarean rates (60.22% versus 31.21%; OR 3.34; 95% CI, 3.14–3.55; *p* < 0.001).

**Conclusion** Hypertensive disorders are associated with higher rates of cesarean deliveries and higher risk of preterm infants, low birthweight infants and a higher risk of fetal deaths.

## Resumo

**Objetivo** Avaliar a prevalência dos distúrbios hipertensivos, resultados perinatais (recém-nascidos pré-termo, recém-nascidos de baixo peso, índice de Apgar < 7 no 5° minuto e óbitos fetais) e as taxas de cesarianas nas gestantes internadas para assistência ao parto na Maternidade Hilda Brandão da Santa Casa de Belo Horizonte, Belo Horizonte, MG, Brasil, no período de 1° de março de 2008 a 28 de fevereiro de 2018.

**Métodos** Foi realizado um estudo analítico, observacional, longitudinal. Os grupos selecionados para comparação foram gestantes com e sem distúrbios hipertensivos. Do total de 36.724 gestantes, 4.464 foram diagnosticadas com distúrbios hipertensivos e 32.260 não apresentavam distúrbios hipertensivos.

**Resultados** A prevalência dos distúrbios hipertensivos foi de 12,16%; Os resultados perinatais e as taxas de cesarianas entre os 2 grupos de gestantes com e sem distúrbios hipertensivos foram: recém-nascidos pré-termo (21,70% *versus* 9,66%; odds ratio [OR] 2,59; intervalo de confiança [IC] 95%, 2,40–2,80; *p* < 0,001); recém-nascidos de baixo peso (24,48% *versus* 10,56%; OR 2,75; IC 95%, 2,55–2,96; *p* < 0,001); índice de Apgar < 7 no 5° minuto (1,40% *versus* 1,10%; OR 1,27; IC 95%, 0,97–1,67; *p* = 0,084); fetos mortos diagnosticados previamente ao parto (1,90% *versus* 0,91%; OR 2,12; IC 95%, 1,67–2,70; *p* < 0,001); taxas de cesarianas (60,22% *versus* 31,21%; OR 3,34; IC 95%, 3,14–3,55; *p* < 0,001).

**Conclusão** Os distúrbios hipertensivos estão associados a maiores taxas de cesarianas, ao maior risco de recém-nascidos pré-termo, recém-nascidos de baixo peso e a um maior risco de óbitos fetais.

## Introduction

Hypertensive disorders complicate up to 10% of all pregnancies and are one of the main causes of maternal and perinatal morbidity and mortality, besides playing a key role in prematurity. 1 2 3 Hypertensive disorders are classified into four categories: 1) pre-eclampsia (PE)-eclampsia, 2) chronic hypertension (of any etiology), 3) chronic hypertension with superimposed PE and 4) gestational hypertension. 1 4 5 6 Pre-eclampsia affects 2 to 8% of pregnant women. 6 The main maternal complications in PE are eclampsia, coagulopathy (disseminated intravascular coagulation), stroke, pulmonary edema, severe renal failure, liver infarction or hemorrhage, myocardial infarction, retinal injury, placental abruption and death. 1 5 6 7 8 Eclampsia affects ∼ 3.2% of patients suffering from PE with severe features. 6 9 It occurs in a ratio of 1/2000 deliveries in developed countries and from 1/100 to 1/1,700 deliveries in developing countries. 9 HELLP syndrome (H: hemolysis; EL: elevated liver enzymes; LP: low platelet) is associated with high rates of maternal morbidity and mortality. 1 6 10 11 Its occurrence is ∼ 1 to 2% in patients with PE with severe features. 12 In PE, placental ischemia may lead to fetal growth restriction and placental abruption with a subsequent increased risk of prematurity. Other complications are perinatal death and hypoxia-related neurological injuries. 1 6 13 14 Induction of labor can be performed as long as fetal well-being is assured and maternal clinical conditions allow. 15 16 In cases of gestational hypertension and PE without severe features, pregnancy should be followed-up with maternal and fetal assessment, and delivery can be scheduled for the 37 ^th^ week of pregnancy. 17 18 Immediate termination of pregnancy, once diagnosed after 36 weeks, is related to a reduction in the risk of PE with severe features, HELLP syndrome, eclampsia, pulmonary edema and placental abruption, when compared with pregnant women with pregnancy management beyond 36 weeks, neither leading to increased neonatal morbidity nor higher c-section rates. 18 Gestational hypertension may progress, in almost half of the cases, to PE. 1 4 6 In cases of gestational hypertension or PE with severe features, delivery is recommended when the diagnosis is done at or beyond the 34 ^th^ week. 19 Chronic hypertension (CH) affects up to 5% of pregnant women. 1 Maternal risks related to CH such as maternal mortality, stroke, pulmonary edema or renal failure are 5 to 6 times higher than in normotensive women. 20 21 The risk of gestational diabetes is also increased. 22 Increased prematurity is directly related to the indication for termination of pregnancy, with a 2-fold higher incidence of fetal growth restriction when compared with women not affected by CH. 23 Chronic hypertension with superimposed PE is characterized by the onset of PE in hypertensive patients prior to pregnancy. It affects from 20 to 50% of chronically hypertensive patients. 20 24 25

## Cesarean Section Rates

The number of cesarean sections has been increasing over the years around the world. Global rates increased from 12.1% in 2000 to 21.1% in 2015. 26 In Brazil, in 2010, c-section rates reached as high as 52.33% of all deliveries and 55.43%, in 2016. 27 The mode of delivery in patients with hypertensive disorders will depend on maternal and fetal clinical conditions as well as on gestational age. 6

## Preterm Infant

Prematurity is the main cause of neonatal morbidity and mortality. Preterm infants are those born between the 20 ^th^ and before the full 37 weeks of gestation, in other words, 36 weeks and 6 days. 28 Considering 2010 worldwide figures, 15,000,000 out of 135,000,000 newborns were preterm, accounting for 11.1% of all births. 29 In Brazil, preterm birth rates in 2016 reached up to 11.34%. 27

## Low Birthweight Infant

Newborns < 2,500 g are already considered low weight. 28 About 20 million low birthweight and premature newborns are born annually around the world, and one third die before reaching the age of 1 year. 30 In Brazil, perinatal conditions such as birth asphyxia, infections and respiratory problems are the main causes of infant mortality and are more frequent in preterm and low birthweight infants. 30

## APGAR Score

The APGAR score was described by Dr. Virginia Apgar in 1953 and is a tool for classifying the clinical condition of newborns soon after birth and assessing the effectiveness of resuscitation measures whenever necessary. 31 The persistence of a low APGAR score at the 5 ^th^ minute indicates the need for further therapeutic efforts and the severity of the underlying problem of the baby. If the 5-minute APGAR score is > 6, perinatal asphyxia is rather unlikely to happen. 32 33 The present study aimed to evaluate the prevalence of hypertensive disorders, perinatal outcomes and the cesarean rates in pregnant women hospitalized for delivery at the Maternidade Hilda Brandão da Santa Casa de Belo Horizonte, Belo Horizonte, state of Minas Gerais, Brazil.

## Methods

### Study Design and Participants

An analytical, observational, retrospective, longitudinal, case-control study was performed. The groups selected to be compared were pregnant women with and without hypertensive disorders admitted to the hospital for delivery from March 1st, 2008 to February 28, 2018 at the Maternidade Hilda Brandão da Santa Casa de Belo Horizonte, state of Minas Gerais, Brazil. Out of the 43,775 admissions performed, 36,724 were pregnant women admitted for delivery. A total of 32,260 of all women admitted for childbirth had no hypertensive disorders, and 4,464 were diagnosed with hypertensive disorders. In the 10-year period of the study (March 1, 2008 to February 28, 2018), 740 pregnancies were multifetal gestations, with an incidence of 2.02% in the general population, of 1.95% in pregnant women without hypertensive disorders and of 2.49% in pregnant women with hypertensive disorders. Due to multifetal gestations, the 36,724 pregnant women admitted for delivery gave birth to 37,464 newborns. The 32,260 pregnant women without hypertensive disorders hospitalized for delivery care gave birth to 32,889 newborns. The 4,464 pregnant women with hypertensive disorders hospitalized for delivery care gave birth to 4,575 newborns.

### Outcomes Measures

The primary outcome was the prevalence of hypertensive disorders and the secondary outcomes were c-section rates and perinatal outcomes (APGAR score < 7 at 5 minutes, low birthweight (LBW) infants, preterm infants and stillbirths) in pregnant women admitted to the hospital with and without hypertensive disorders for delivery care.

### Inclusion and Exclusion Criteria

All patients admitted for delivery care (36,724 patients) were included in the present study. Patients admitted for other procedures (clinical treatment, uterine curettage, laparotomies, cerclages and other procedures) were excluded from the study.

### Ethical Protocol

Informed consent was waived by the Research Ethics Committee of the Santa Casa de Belo Horizonte given the fact that the analysis of the statistical database was performed after the patients were discharged from the hospital, with no nominal identification. The present study was approved by the Research Ethics Committee of the Santa Casa de Belo Horizonte (under the number CAAE: 85213418.3.0000.5138).

## Statistical Analysis

The statistical methodology used was the two proportions Z-test for comparisons, with a 5% significance level. Therefore, *p-values* < 0.05 were considered statistically significant.

## Results

**Fig. 1:**
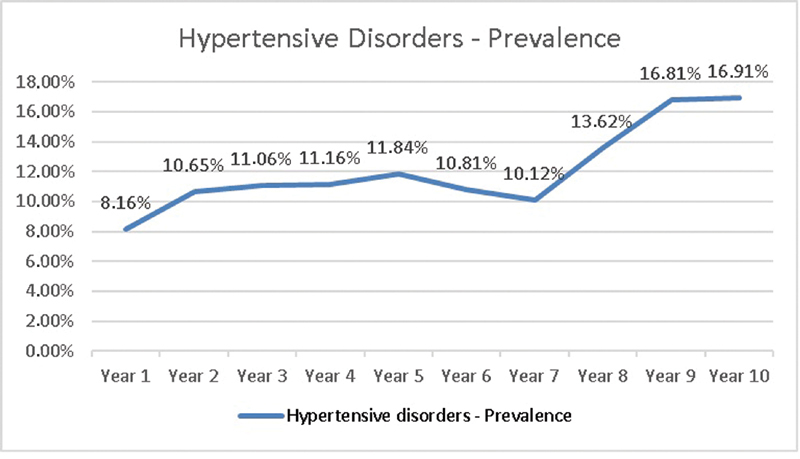
Year on year prevalence of hypertensive disorders at the Maternidade Hilda Brandão.

A total of 36,724 pregnant women were admitted for delivery at the Maternidade Hilda Brandão da Santa Casa de Belo Horizonte, between March 1st, 2008 and February 28, 2018. A total of 32,260 of those had no hypertensive disorders, and 4,464 were diagnosed with hypertensive disorders. The rates of preterm infants, low birthweight infants, APGAR < 7 at 5 minutes and stillbirths (identified prior to delivery) in the general population were 11.13%, 12.25%, 1.14% and 1.03% respectively. A total of 12,775 c-sections were performed out of the 36,724 deliveries, corresponding to 34.73% of all cases. The prevalence of hypertensive disorders in patients admitted for delivery was 12.16%, with 8.16% in the 1 ^st^ year and 16.91% in the 10 ^th^ year ( Fig. 1 ).

**Fig. 2:**
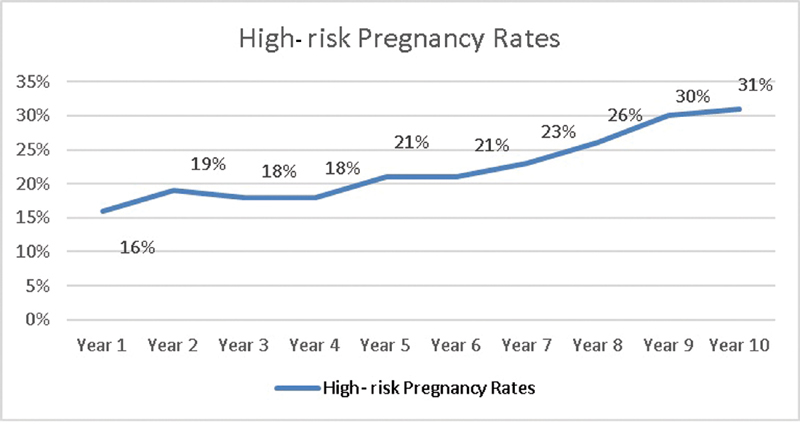
Year on year prevalence of high-risk pregnancies at the Maternidade Hilda Brandão.

The increased prevalence over the years was a consequence of the higher number of high-risk pregnancy patients referred to the Maternidade Hilda Brandão ( Fig. 2 ).

**Fig. 3:**
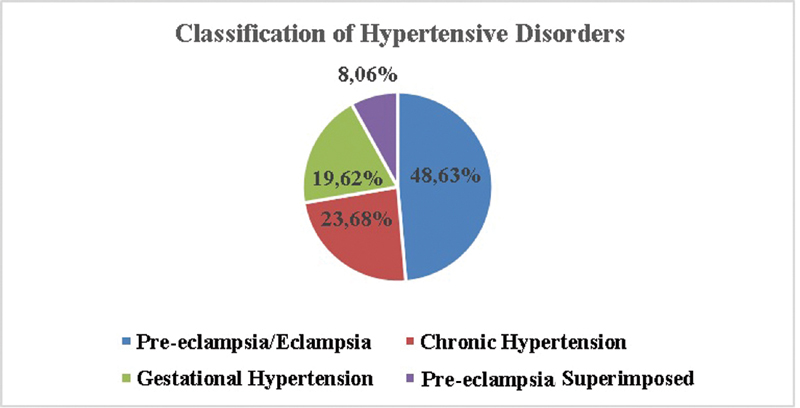
Classification of hypertensive disorders at the Maternidade Hilda Brandão.

Considering all 4,464 pregnant women (diagnosed with hypertensive disorders), 2,171 (48.63%) were classified as PE-eclampsia; 1,057 (23.68%) were classified as CH; 876 (19.62%) were classified as gestational hypertension, and 360 (8.06%) were classified as CH with superimposed PE ( Fig. 3 ).

Throughout the study period, there were 2,531 cases of PE (2,171 women classified as PE-eclampsia and 360 women classified as CH with superimposed PE), with a prevalence of 6.89%, and 876 cases of gestational hypertension, with a prevalence of 2.39%. There were 36 cases of eclampsia, 1 case for every 1,020.1 deliveries, or 1 case for every 70.3 women with PE, with an incidence of 1.42%. There were 115 cases of HELLP syndrome, 105 cases in patients classified as PE/eclampsia and 10 cases in patients classified as CH with superimposing PE, with an incidence of 4.54%. There were 1,417 cases of CH (1,057 women classified as CH and 360 classified as CH with superimposed PE), with a prevalence of 3.86%. Of the 1,417 pregnant women with CH, 360 had superimposed PE, affecting 25.41% of women with CH. Cesarean section rates in pregnant women with hypertensive disorders were 60.22%, and in the control group 31.21%; OR 3.34; 95% CI, 3.14–3.55; *p* < 0.001 ( Table 1 ).

**Table 1:** Cesarean section rates at the Maternidade Hilda Brandão

	Pregnant With Hypertension		Pregnant Without Hypertension		*p-value* *		OR	95%CI
	n	(%)	n	(%)				
C Sections	2,688	(60.22)	10,068	(31.21)	< 0.001		3.34	3.14–3.55
Total deliveries	4,464		32,260					

Abbreviations: CI, confidence interval; OR, odds ratio.

* Proportion Comparison Z Test.

The rate of preterm infant in pregnant women with hypertensive disorders was 21.7%, and in the control group, 9.66%; OR 2.59; 95% CI, 2.40–2.80; *p* < 0.001 ( Table 2 ).

**Table 2:** Preterm infant rates at the Maternidade Hilda Brandão

	Pregnant With Hypertension		Pregnant Without Hypertension		*p-value* *	OR	95%CI
	n	(%)	n	(%)			
Preterm Infant	993	(21.70)	3,178	(9.66)	< 0.001	2.59	2.40–2.80
Total Infants	4,575		32,889				

Abbreviations: CI, confidence interval; OR, odds ratio.

* Proportion Comparison Z Test.

The rate of low birthweight (LBW) infants in pregnant women with hypertensive disorders was 24.48%, and in the control group it was 10.56%; OR 2.75; 95% CI, 2.55–2.96; *p* < 0.001 ( Table 3 ).

**Table 3:** Low birthweight infant rates at the Maternidade Hilda Brandão

	Pregnant With Hypertension		Pregnant Without Hypertension		*p-value* *	OR	95%CI
	n	(%)	n	(%)			
LBW Infant	1,120	(24.48)	3,472	(10.56)	< 0.001	2.75	2.55–2.96
Total Infants	4,575		32,889				

Abbreviations: CI, confidence interval; LBW, low birthweight; OR, odds ratio.

* Proportion Comparison Z Test.

The rate of infants with APGAR < 7 at the 5th minute in pregnant women with hypertensive disorders was 1.40% and 1.10% in the control group; OR 1.27, 95% CI, 0.97–1.67; *p* < 0.001 ( Table 4 ).

**Table 4:** APGAR < 7 at 5th minute rates at the Maternidade Hilda Brandão

	Pregnant With Hypertension		Pregnant Without Hypertension		*p-value* *	OR	95%CI
	n	(%)	n	(%)			
APGAR < 7	63	(1.40)	360	(1.10)	0.084	1.27	0.97–1.67
Total Infants	4,488		32,591				

Abbreviations: CI, confidence interval; OR, odds ratio.

* Proportion Comparison Z Test.

The dead fetuses diagnosed prior to delivery were excluded from the APGAR analysis (385 in the general population, or 1.03%), with 87 (1.90%) out of the total number of newborns in pregnant women with hypertension and 298 (0.91%) out of the total number of newborns in pregnant women without hypertension; OR 2.12; 95% CI, 1.67–2.70; *p* < 0.001 ( Table 5 ).

**Table 5:** Dead fetuses rates before delivery at the Maternidade Hilda Brandão

	Pregnant With Hypertension		Pregnant Without Hypertension		*p-value* *	OR	95%CI
	n	(%)	n	(%)			
Dead fetuses	87	(1.90)	298	(0.91)	< 0.001	2.12	1.67–2.70
Total Infants	4,575		32,889				

Abbreviations: CI, confidence interval; OR, odds ratio.

* Proportion Comparison Z Test.

## Discussion

The Maternidade Hilda Brandão is a reference in high-risk pregnancy in the state of Minas Gerais, Brazil. It was founded on June 24, 1916, being the first maternity hospital in Belo Horizonte. Over the following 10-year period (March 1, 2008 to February 28, 2018), 36,724 deliveries were performed at an average of 306.03 per month. The present study revealed the existence of a statistically significant difference between the proportion of c-sections, preterm infants, LBW infants, and dead fetuses (diagnosed before delivery) for pregnant women with and without hypertensive disorders. It was also shown that there is no significant difference between the proportion of newborns with APGAR score < 7 at the 5 ^th^ minute for pregnant women affected or not by hypertensive disorders. Hypertensive disorders complicate up to 10% of all pregnancies. 1 In the present study, the prevalence of hypertensive disorders increased from 8.16% in the 1 ^st^ year to 16.91% in the 10 ^th^ year, with an annual average of 12.16%. High-risk pregnancy (HRP) rates increased from 16% in the first year of the study to 31% in the last year, which justifies the increased prevalence of hypertensive disorders. Pre-eclampsia affects 2 to 8% of pregnant women and CH affects up to 5%. 1 6 In the present study, the prevalence of PE was 6.89%, and 3.86% for CH. The prevalence of gestational hypertension was 2.39%. Eclampsia affects ∼ 3.2% of patients with PE with severe features. 6 9 It occurs in a ratio of 1/100 to 1/2,000 deliveries. 9 At the Maternidade Hilda Brandão, there were 36 eclampsia cases; 1 per 1,020.1 deliveries; 1 per 70.3 patients with PE, with an incidence of 1.42%. The occurrence of HELLP syndrome is of ∼ 1 to 2% in patients with PE with severe features. 12 At the Maternidade Hilda Brandão, there were 115 HELLP syndrome cases, with an incidence of 4.54%. Chronic hypertension with superimposed PE affects from 20 to 50% of chronically hypertensive patients. 20 24 25 In the present study, superimposed PE affected 25.41% of the patients with CH.

## Conclusion

Hypertensive disorders are associated with a higher proportion of c-section deliveries, preterm newborns and LBW infants. The proportion of dead fetuses before hospital admission for delivery is also higher in pregnant women with hypertensive disorders. Therefore, good prenatal care is essential to prevent fetal death before hospital admission for delivery. The evaluation of newborns by the APGAR score at 5 minutes showed no significant differences between the proportion of newborns with APGAR < 7 at 5 minutes for pregnant women with and without hypertensive disorders, which leads to the understanding that adequate and timely delivery assistance is critical to good fetal conditions at birth.
